# Disrupted H_2_S Signaling by Cigarette Smoking and Alcohol Drinking: Evidence from Cellular, Animal, and Clinical Studies

**DOI:** 10.3390/antiox10010049

**Published:** 2021-01-03

**Authors:** Ethan Read, Jiechun Zhu, Guangdong Yang

**Affiliations:** 1 Department of Chemistry and Biochemistry, Laurentian University, 935 Ramsey Lake Road, Sudbury, ON P3E 2C6, Canada; eread@laurentian.ca; 2 Cardiovascular and Metabolic Research Unit, Laurentian University, 935 Ramsey Lake Road, Sudbury, ON P3E 2C6, Canada; jzhu5@laurentian.ca; 3 Department of Biology, Laurentian University, 935 Ramsey Lake Road, Sudbury, ON P3E 2C6, Canada

**Keywords:** H_2_S, cigarette smoking, alcohol drinking, oxidative stress, inflammation

## Abstract

The role of endogenous hydrogen sulfide (H_2_S) as an antioxidant regulator has sparked interest in its function within inflammatory diseases. Cigarette and alcohol use are major causes of premature death, resulting from chronic oxidative stress and subsequent tissue damage. The activation of the Nrf2 antioxidant response by H_2_S suggests that this novel gasotransmitter may function to prevent or potentially reverse disease progression caused by cigarette smoking or alcohol use. The purpose of this study is to review the interrelationship between H_2_S signaling and cigarette smoking or alcohol drinking. Based on the databases of cellular, animal, and clinical studies from Pubmed using the keywords of H_2_S, smoking, and/or alcohol, this review article provides a comprehensive insight into disrupted H_2_S signaling by alcohol drinking and cigarette smoking-caused disorders. Major signaling and metabolic pathways involved in H_2_S-derived antioxidant and anti-inflammatory responses are further reviewed. H_2_S supplementation may prove to be an invaluable asset in treating or preventing diseases in those suffering from cigarette or alcohol addiction.

## 1. Introduction

Cigarette smoking and alcohol consumption are the leading causes of the global burden of disease and are major contributors to premature mortality in industrial countries. Cigarette smoking and alcohol drinking can cause damage in nearly all body organs, especially cardiovascular, nervous, hepatic, gastrointestinal and pulmonary systems. Systemic disease caused by cigarettes or alcohol use can be acute or chronic [1,2]. Chronic obstructive pulmonary disease (COPD), alcoholic fatty liver disease, cardiomyopathy, neurodegeneration, gastric ulceration, atherosclerosis, fetal alcohol spectrum disorder, low birth weight, and many forms of cancer are all can be caused by cigarette smoking, alcohol drinking, or both [3,4,5,6]. Concurrent consumption of alcohol and cigarette smoking further increase the risk of developing these diseases [7]. Treatment of these diseases has been progressing, with some focusing on ameliorating symptoms in late stage disease, but the most effective treatment is abstinence from using cigarettes and alcohol [8,9].

**Figure 1:**
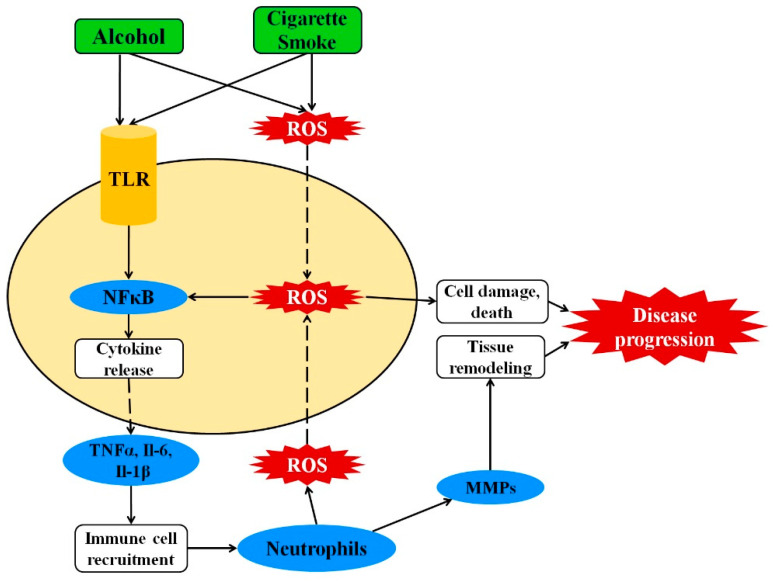
Cigarette smoking and alcohol drinking induce the oxidative and inflammatory response. Exposure to cigarette or alcohol leads to oxidative stress and stimulation of NFκB. NFκB promotes secretion of proinflammatory cytokines TNFα, IL-6, and IL-1β, which then strengthen immune cell recruitment. Recruited neutrophils produce additional ROS and release metalloproteases, which induce cell damage and matrix degradation, respectively. The net result of constitutive activation of inflammatory pathways is tissue destruction and disease progression. Abbreviations: NFκB, nuclear factor kappa-light-chain-enhancer of activated B cells; TLR, toll-like receptor; TNFα, tumor necrosis factor α; IL, interleukin; ROS, reactive oxygen species.

The pathology of cigarette smoking and alcohol drinking-caused diseases is generally attributed to excessive oxidative stress and chronic inflammation [10,11]. The primary effector of disease is oxidative imbalance caused by the rapid accumulation of reactive oxygen species (ROS) within the cells. Cigarettes introduce ROS via a multitude of cytotoxic compounds, including nicotine, aldehydes, heavy metals, polycyclic aromatic hydrocarbons, and more, while alcohol introduces oxidative stress through the metabolism of ethanol to acetaldehyde [12,13]. High level of oxidative stress triggers NFκB-mediated release of proinflammatory cytokines, such as TNF-α and interleukins, which consequently recruit the immune system to the affected tissues [14,15,16]. Recruited neutrophils induce further oxidative stress through the production of ROS, and the secretion of matrix metalloproteases for degradation of extracellular matrix [17,18]. Constitutive oxidative stress results in cell death, and the repeated destruction and reconstruction of the extracellular matrix eventually leading to fibrosis. These effects cumulatively induce organ dysfunction and tissue death (Figure 1).

## 2. H_2_S as a Novel Gasotransmitter

Instantly recognizable by its distinct rotten-egg smell, hydrogen sulfide (H_2_S) has traditionally been viewed as nothing more than an unpleasant smelling and toxic gas. At high concentrations this certainly holds true, as H_2_S prevents cellular respiration by binding with and inhibiting cytochrome C oxidase [19]. During the past decades, however, a different story has emerged. First discovered as an endogenous molecule in the 1960s, the gaseous compound was initially believed to be a metabolic waste product. It was not until the mid-1990s that a physiological role for the so-called waste was uncovered, when it was found that H_2_S activates long-term potentiation in the brain [20]. Since then, H_2_S has been classed as the third gasotransmitter, alongside carbon monoxide and nitric oxide, and a novel field of research has developed around the diverse biological effects of H_2_S [21,22].

**Figure 2:**
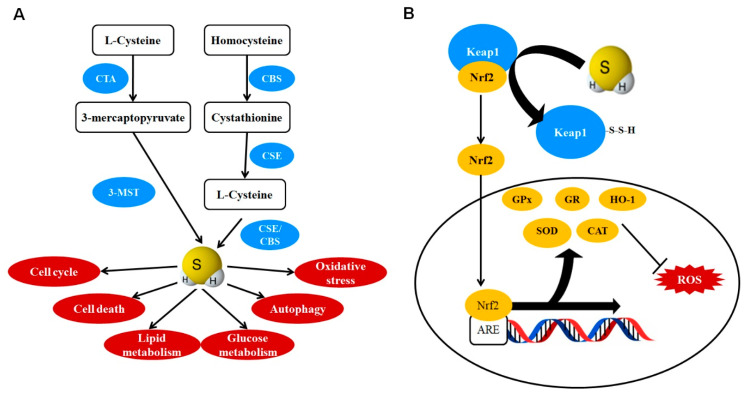
H_2_S is recognized as a novel gasotransmitter. (**A**) Enzymatic H_2_S production mediated by CSE, CBS, and 3MST/CAT. Endogenously produced H_2_S regulates diverse cellular functions, including cell cycle, cell death, lipid metabolism, glucose metabolism, oxidative stress, and autophagy. (**B**) H_2_S mediates its antioxidant effects through activation of Keap1/Nrf2. S-sulfhydration of cysteine residues on Keap1 causes the release of Nrf2, which is then able to translocate into the nucleus and bind with ARE. Transcription of ARE leads to production of several AOEs, which collectively reduce oxidative stress. Abbreviations: 3-MST, 3-mercaptopyruvate sulfurtransferase; ARE, antioxidant response element; AOE, antioxidant enzyme; CAT, catalase; CBS, cystathionine β-synthase; CSE, cystathionine γ-lyase; CTA, cysteine transaminase; GPx, glutathione peroxidase; GR, glutathione reductase; H_2_S, hydrogen sulfide; Keap1, Kelch-like ECH associated protein 1; Nrf2, nuclear factor erythroid 2-related factor 2; SOD, superoxide dismutase; ROS, reactive oxygen species.

The endogenous production of H_2_S is well regulated, and at least three distinct enzymatic pathways contribute to endogenous production of H_2_S in various tissues. The three pathways are denoted by the primary enzyme involved, those being cystathionine γ-lyase (CSE), cystathionine β-synthase (CBS), and 3-mercaptosulfur transferase (3MST) (Figure 2A) [23,24]. CBS is the most prominent within the brain, while CSE plays the largest role in the other major tissues and 3MST plays a secondary or tertiary role in most tissues [23].

The physiological functions of H_2_S include regulation of cellular senescence, cell cycle, metabolism, vasorelaxation, autophagy, and oxidative stress (Figure 2A) [25,26,27]. H_2_S post-translational modification of protein by S-sulfhydration mediates most of the cellular functions [28,29]. S-sulfhydration consists of the formation of a persulfide group (-SSH) or polysulfide chains on active cysteine residues within a protein [30,31]. These changes result in structural and conformational alterations, eventually changing protein function and catalytic ability [32]. The ubiquity of cysteine residues in proteins, ranging from structural disulfide bridges to catalytic sites, ensures that H_2_S has wide ranging effects on many proteins [28,33]. There is some doubt on the direct formation of S-sulfhydration in free cysteine by H_2_S [28]. Sulfane sulfur, a group of sulfur-containing compounds, may interact with oxidized cysteine in target protein resulting in formation of S-sulfhydration [29,34]. The generation of cysteine S-sulfhydration by sulfane sulfur depend on the location and oxidative status of individual cysteine residues [28,34]. S-sulfhydration is not only a post-translational modification of protein. Akaike et al. proved that cysteine S-sulfhydration can also occur at the translational stage with the aid of cysteinyl tRNA synthetase [35]. In contrast, thioredoxin 1, an important reducing enzyme that cleaves disulfides in proteins, facilitates protein S-desulfhydration [36]. Future studies need to explore the regulatory mechanisms of cysteine S-sulfhydration formation and map the global protein S-sulfhydrome in both health and disease. 

The most notable target for S-sulfhydration is the regulation of the Nrf2/Keap1 system. Nuclear factor (erythroid-derived 2)-like factor 2 (Nrf2) is a master regulator of the antioxidant response, which translocates to the nucleus and binds with the antioxidant response element (ARE) [37]. Increased ARE transcription stimulates several antioxidant enzymes (AOEs), including superoxide dismutase, glutathione peroxidase, catalase, and heme oxygenase-1 [38]. These enzymes collectively act to maintain oxidative homeostasis and reduce ROS-mediated damage within the cells. In the cytoplasm, Nrf2 is bound by Kelch-like ECH-associated protein 1 (Keap1), which promotes the ubiquitination and degradation of Nrf2 by the proteasome [37]. S-sulfhydation of Keap1 prevents binding to Nrf2, allowing for Nrf2 nuclear localization and AOE transcription, thus making H_2_S as an activator of the Nrf2 antioxidant master switch (Figure 2B) [27]. In addition to increasing transcription of AOE, H_2_S can directly reduce ROS levels by restoring glutathione levels and/or through chemical reduction of offending molecules [39,40].

**Figure 3:**
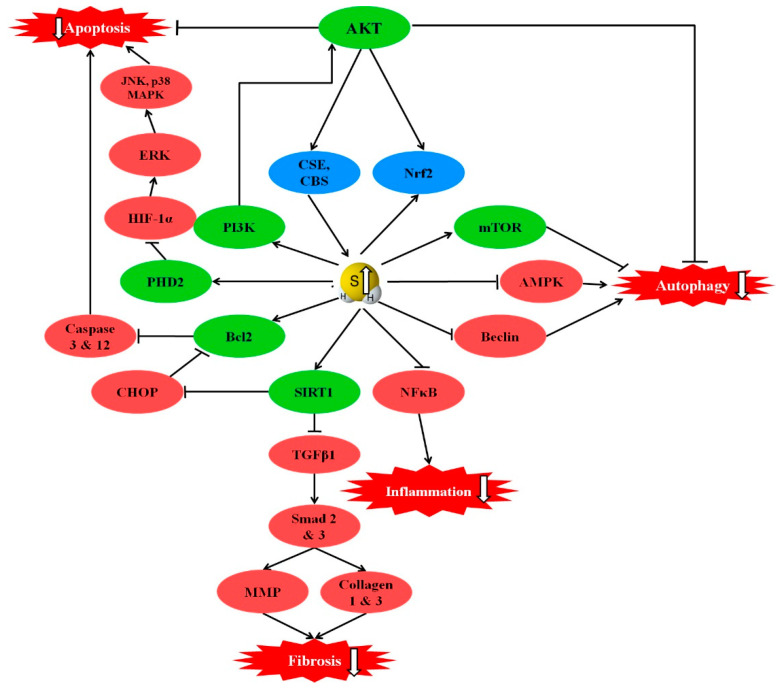
The signaling pathways underlying H_2_S regulation of inflammation, apoptosis, fibrosis, and autophagy. Abbreviations: Bcl2, B-cell lymphoma 2; CHOP, C/EBP homologous protein; CBS, cystathionine β-synthase; CSE, cystathionine γ-lyase; ERK, extracellular signal-regulated kinase; HIF-1α, hypoxia-inducible factor 1-alpha; JNK, c-Jun N-terminal kinase; MAPK, mitogen-activated protein kinase; MMP, matrix metallopeptidase; mTOR, mammalian target of rapamycin; TGFβ1, transforming growth factor beta 1.

Endogenous H_2_S likely play a major role in the innate defense against oxidative stress and inflammation-mediated disease (Figure 3) [41]. Endogenous H_2_S is influenced by many factors, including genetic, dietary, and environmental components. Major dietary sources of sulphur include cruciferous vegetables, such as broccoli or kale, and garlic. These foods contain many organosulphur compounds, which are readily metabolized by the body to yield H_2_S, and are often considered H_2_S donors as a result [42]. Regular exercise boosts endogenous H_2_S production [43], while ”unhealthy” habits such as high-fat diets and sedentary lifestyle decrease H_2_S level due to lower expressions of H_2_S-generating genes [44].

Despite a large amount of H_2_S research in both health and disease having been published, a comprehensive review on the relationship between H_2_S signaling and smoking or drinking is lacking. Given the importance of H_2_S in biology and medicine, this review article summarizes the recent research progress on the involvement of H_2_S signaling in cell damage and organ dysfunction related to cigarette smoking and alcohol drinking. Based on the databases from Pubmed, “H_2_S and smoking”, “H_2_S and cigarettes”, “H_2_S and tobacco”, “H_2_S and alcohol”, and “H_2_S and ethanol” were used as phrases to search literature related to H_2_S signaling in biology and medicine. The publications related to environmental pollution or H_2_S toxicology were excluded from this review. 

## 3. H_2_S and Cigarette Smoking

### 3.1. Clinical Observations

Definitive links between smoking and the changes in H_2_S levels in human have been uncovered. Serum H_2_S level was significantly lower in smokers than nonsmokers [45]. The expression of CSE was decreased in smokers’ lung tissue as detected by immunohistochemistry and Western blotting [46]. Chronic obstructive pulmonary disease (COPD) is a chronic inflammatory disease and can be caused by long-term exposure to cigarette smoking. Serum H_2_S is elevated in the patients with stable COPD, yet is lower in control smokers and those suffering from acute exacerbation of COPD (AE-COPD) [46]. In contrast, sputum H_2_S levels in AE-COPD patients were higher than those with stable COPD [47]. It was further found that exhaled H_2_S levels were similar in patients with AE-COPD, stable COPD, and healthy controls, while COPD patients without eosinophilia had significantly higher levels of exhaled H_2_S compared with subjects with eosinophilia [48,49]. These findings point that disrupted H_2_S signaling might be valuable marker to examine the different status of COPD. More excitingly, after undergoing a 10-day cycle of sulphurous thermal water inhalation, a reduction of the inflammatory activity was observed in a large number of heavy current and former smokers, suggesting the potential benefits of H_2_S for smokers [50]. Moreover, Bates et al. provided some epidemiologic evidence that ambient H_2_S exposures may benefit lung function, possibly through airway smooth muscle relaxation [51]. Maternal smoking during pregnancy increases the risk of asthma and borderline personality disorder-like symptoms in the progeny. A recent study further found that the expressions of three H_2_S-generating genes, including CSE, CBS, and 3MST, were all lower in the placentas from mothers who smoked during pregnancy in comparison with the mothers who never smoked. Quitting smoking during the first trimester of pregnancy could partially improve the expressions of these H_2_S-generating genes in the placentas [52]. More studies need to be conducted to validate if the downregulated expressions of these H_2_S-generatiang enzymes might be a biomarker for asthma susceptibility in children. 

### 3.2. Animal Studies

Exposure of laboratory animals to cigarette smoking can reproduce some morphologic and physiologic manifestations of diseases as happened in humans. Several rodent models have been employed in the study of H_2_S signaling and cigarette smoking-induced organ damage. The results obtained in these preclinical animal studies demonstrated that H_2_S signaling was often disrupted by cigarette smoking exposure, while exogenously applied H_2_S was able to provide beneficial effects against cigarette smoking exposure-caused pulmonary and vascular damage, as well as abnormal development of natal offspring (Table 1). 

#### 3.2.1. H_2_S Protects from Maternal/Prenatal Cigarette Smoking Exposure-Induced Damage

Maternal cigarette smoking exposure has been found to increase oxidative stress, mitochondrial dysfunction, and cell apoptosis in offspring. Zhang et al. first observed that cigarette smoking exposure to rats during pregnancy blunted hypercapnic respiratory responses in the neonates, which could be relieved by H_2_S pretreatment [53]. The same group further reported that H_2_S protected the offspring from apoptosis and helped the recovery of central chemoreception via the activation of mitoK_ATP_ channels [54]. It was proposed that H_2_S may have potential therapeutic value for maternal cigarette smoking exposure-induced central chemoreception deficit-related diseases, such as sudden infant death syndrome.

Prenatal cigarette smoking exposure is known to induce respiratory problems in infants. The mRNA and protein expression of 3MST was upregulated in the neurons of medullary respiratory nuclei of neonatal rats by intrauterine cigarette smoking exposure, indicating that endogenous H_2_S derived from 3MST may protect medullary respiratory centers against cigarette smoking-caused injury [55]. Wang et al. found that cigarette smoking exposure caused a more slowly and deeply breathing in rat neonates under sevoflurane anesthesia, while pretreatment with H_2_S via intraperitoneal injection NaHS alleviated sevoflurane-induced respiratory suppression [56]. Mechanistically, H_2_S was also able to prevent the increase in the malondialdehyde level, upregulation of inflammatory response, and suppression of antioxidase activity in the neonatal rats induced by prenatal cigarette smoking exposure [57,58]. H_2_S was also able to improve cigarette smoking-caused fetal and placental development. In an animal model of cigarette smoking exposure in pregnant rats, the administration of NaHS normalized the reduced fetal and placental weights by cigarette smoking [59]. Further studies demonstrated that H_2_S relieved cigarette smoking-induced abnormalities of junctional and labyrinthine zones, and ultrastructural alterations in rat placenta [60].

In another study, mice were exposed to secondhand cigarette smoking throughout the gestational period, and then H_2_S biogenesis in lungs from the pups of the F1 and F2 generation were analyzed. It was found that mouse lung exposed gestationally to cigarette smoking had significantly lower levels of CSE, CBS, and 3MST in both the F1 and F2 generation. Moreover, the lungs from F1 and F2 progenies displayed with impaired angiogenesis and alveolarization, suggesting that a lower level of H_2_S is correlated with the increased risk of lung disorders [52]. 

#### 3.2.2. H_2_S Attenuates Cigarette Smoking-Induced Lung Damage

Long-term cigarette smoking exposure can induce airway remodeling, thus damaging the respiratory function. By using a mouse model of cigarette smoking exposure, Guan et al. found that treatment with NaHS significantly attenuated airway thickening and collagen deposition, as characterized by decreased expressions of α-SMA and collagens and myofibroblast accumulation [61]. The protective role of H_2_S against airway remodeling was attributed to its inhibition of SIRT1-mediated TGF-β1/Smad3 activation and epithelial–mesenchymal transition. Han et al. also reported that NaHS ameliorated cigarette smoking-induced thickness of bronchial walls and emphysema development in mice via AKT/Nrf2 pathways [62]. With a rat model of cigarette smoking exposure, Wang et al. demonstrated that H_2_S inhibited cigarette smoking-caused oxidative stress, inflammation, and airway remodeling through inhibition of the TGF-β1/Smad pathway [63]. 

CSE deficiency abolished endogenous H_2_S generation in mouse lung and promoted the secretion of cytokines and chemokines in bronchoalveolar lavage fluid following cigarette smoking exposure and respiratory syncytial virus infection [64]. These data suggest that the lower level of endogenous H_2_S due to immature H_2_S-genarating enzymes could cause more severe manifestations of virus infection under cigarette smoking exposure or other stress conditions. In one study, blockage of endogenous H_2_S generation by propargylglycine (PPG), a CSE inhibitor, however, did not worsen the detrimental effect of cigarette smoking exposure on pulmonary remodeling, excluding the involvement of CSE-derived H_2_S in lung protection [65]. In contrast, other studies demonstrated that PPG aggravated lung pathology score and increased airway reactivity in cigarette smoking-exposed rats, suggesting that endogenous H_2_S may have a protective role of anti-inflammation and bronchodilation in chronic cigarette smoking-induced pulmonary injury [66,67,68]. The discrepancy may be due to the dose and time of cigarette smoking exposure and PPG administration, as well as the age of rats used in these studies. 

#### 3.2.3. H_2_S Ameliorates Cigarette Smoking Exposure-Induced Cardiac Damage

Smoking is a risk factor causing cardiovascular disease, and about a third of smoking-related deaths result from heart complications. Animal models of cigarette smoking exposure all demonstrated the potential protection of H_2_S against abnormal heart functions. In a rat model of cigarette smoking exposure, CSE expression was downregulated in heart tissues and H_2_S levels in plasma were also lower, and the left ventricular systolic function was remarkably lower. Supplementation of exogenous H_2_S would improve the heart functions. The protective role of H_2_S against cigarette smoking -induced cardiac dysfunction was associated with PI3K/Akt-mediated Nrf2 nuclear translocation and increased expression of antioxidant genes followed by reduced cell apoptosis and autophagy [69,70]. Consistent with these studies, Wiliński et al. also proved that administration of nicotine to mice caused a marked decrease in H_2_S level in heart tissues [71]. In the presence of vitamin D3, nicotine-induced structural disorder in the aortic and carotid arterial wall could be ameliorated by exogenous applied H_2_S [72]. In addition, cigarette smoking exposure lowered CSE.

**Table 1:** Animal models used for analyzing H_2_S signaling and cigarette smoking-induced damage.

Animal Species	Cigarette Smoking (CS) Administration	H_2_S Supplement	Organ Damage	Target Signaling Pathway	Reference
BALB/c mice	6 h/day, 1.52 mg/m^3^ during gestation	N/A	Asthma, bronchopulmonary dysplasia	CSE/CBS, TGFβ1, SOX2	Singh et al. [52]
Sprague–Dawley rats	10 cigarettes/h, twice daily, gestation day 7–20	56 μmole/kg NaHS, daily, 30 min before CS, i.p.	Brainstem, respiratory dysfunction	N/A	Zhang et al. [53]
Sprague–Dawley rats	10 cigarettes/h, twice daily, gestation day 1–20	56 μmole/kg NaHS, daily, 30 min before CS, i.p.	Hypoglossal nerve damage, chemoreception impairment	Caspase/Bax	Lei et al. [54]
Sprague–Dawley rats	10 cigarettes/h, twice daily, gestation day 7–20	56 μmole/kg NaHS, daily, 30 min before CS, i.p.	Decreased respiratory function	N/A	Wang et al. [56]
Sprague–Dawley rats	10 cigarettes/h, twice daily, gestation day 7–20	56 μmole/kg NaHS, daily, 30 min before CS, i.p.	Excitatory synapse disorder, respiratory misregulation	Phox2b, AOE/ROS	Yan et al. [57]
Sprague–Dawley rats	10 cigarettes/h, twice daily, gestation day 7–20	56 μmole/kg NaHS, daily, 30 min before CS, i.p.	Medulla oblongata and hypoglossal nerve damage	AOE, TNFα, IL-6/1β	Yan et al. [58]
C57BL/6J mice	9 filter-tipped cigarettes for 2 h twice daily, 6 days/week	40 mg/kg NaHS via atomization inhalation for 30 min, twice daily	COPD	S1RT/ TGFβ1, AOE/ROS	Guan et al. [61]
C57BL/6 mice	1 puff/min and 10 puffs/cigarette for 1 h daily, 5 days per week for 12 or 24 weeks	50 μmole/kg NaHS, daily, 30 min before CS, i.p.	Emphysema, bronchial remodeling, pulmonary vascular remodeling	TNFα, Nrf2, p-AKT, Caspase	Han et al. [62]
Sprague–Dawley rats	20 cigarettes for 2 h, twice daily, 6 days per week	40 ppm H_2_S exposure, 8 h daily for seven days	COPD	ROS, TNFα/IL6, TGFβ1/Smad	Wang et al. [63]
Sprague–Dawley rats	20 cigarettes for 4 h, 7 days per week	14 μmole/kg NaHS, daily, 30 min before CS, i.p.	COPD, reduced pulmonary response	CSE, TNFα/IL6	Chen et al. [68]
Sprague–Dawley rats	10 cigarettes for 30 min, 4 times daily	14 μmole/kg NaHS, daily, 30 min before CS, i.p.	Left ventricle structure and function damage	CSE/Nrf2, AOE/ROS, PI3K/GS3Kβ	Zhou et al. [69]
Sprague–Dawley rats	10 cigarettes for 30 min, 4 times daily	8 μmole/kg NaHS, daily, i.g.	Left ventricle structure and function damage	Caspase/Bax, JNK/p38, PI3K, Beclin-1, AMPK/mTOR	Zhou et al. [70]
CBA mice	1.5 mg/kg nicotine, daily, i.p.	N/A	Reduced H_2_S production in heart/kidney	N/A	Wiliński et al. [71]
Sprague–Dawley rats	25 mg/kg nicotine, daily, i.g.	56 μmole/kg NaHS, daily, i.p.	Calcification of carotid and aorta	CHOP/CAS, Calponin/SM22α	Li et al. [72]
Sprague–Dawley rats	20 cigarettes, daily, for 30 days	N/A	Vascular constriction	CSE, SUR-2, K_ATP_	Zhang et al. [73]

Expression in rat thoracic aorta and inhibited aortic vascular relaxation, which could in part explain the association between cigarette smoking and disrupted heart functions [73]. Given this evidence, it could be feasible for targeting H_2_S signaling for the prevention and therapy of cigarette smoking-induced vascular disorders.

### 3.3. Cellular Studies

Alveolar epithelial cells in lung are the direct targets for damage by smoking. Exposure to cigarette smoking in human alveolar basal epithelial cells (A549) leads to lower cell viability, higher oxidative stress and cell apoptosis, and increased levels of inflammatory factors, all of which could be attenuated by the addition of NaHS. It was further demonstrated that H_2_S upregulated SIRT1 and protected cigarette smoking-induced mitochondrial dysfunctions in epithelial cells [74]. The following study from the same group showed that H_2_S was also able to inhibit cigarette smoking-induced activation of PHD2/HIF-1α signaling and ERK/JNK/p38 MAPK pathways in alveolar epithelial cells [75]. With bronchial epithelia cells, Lin et al. proved that H_2_S inhibited nicotine-induced morphological changes of apoptosis via inhibition of endoplasmic reticulum stress [76]. These findings suggest that H_2_S has potential therapeutic value in the treatment of cigarette smoking-caused lung damage by directly protecting against epithelial cell death.

The animal studies showed that H_2_S was able to reduce cigarette smoking-induced pulmonary hypertension and vascular remodeling in mice [62]. By using pulmonary artery endothelial cells, Han et al. observed that H_2_S protected against cigarette smoking-induced apoptosis and downregulation of Akt and Nrf2 [62]. The airway smooth muscle cells (ASMCs) are important for controlling airway structure and dilation. The loss of contractile phenotype of ASMCs by cigarette smoking leads to airway hyperresponsiveness and remodeling followed by the development of COPD. Perry et al. reported that H_2_S inhibited proliferation and cytokine release in ASMCs isolated from nonsmokers and smokers, but had less effect on the cells from COPD patients [77]. This points to the role of H_2_S in the stabilization of ASMC phenotype for a novel therapeutic avenue against COPD.

Many studies have reported that cigarette smoking is also a driving factor for periodontitis and bone loss [78,79]. Nicotine, one of the major constitutes in cigarettes, was reported to inhibit osteoblastic differentiation [78]. In contrast, H_2_S was able to promote osteoblastic differentiation in a nicotine-incubated human periodontal ligament cell model via the MAPK/PKC/NF-κB pathways, suggesting the potential of H_2_S against cigarette smoking-induced bone loss [78].

## 4. H_2_S and Alcohol Consumption

### 4.1. Clinical Observations

Clinical evidence about the interaction of H_2_S signaling and alcohol drinking in humans is lacking. One paper reported that H_2_S levels were increased in the breath of chronic alcohol users, suggesting that circulating H_2_S may be elevated by alcohol drinking [80]. More studies need to be investigated to explore whether H_2_S signaling is altered in alcohol users.

### 4.2. Animal Studies

Animal models are often used in the laboratory to mimic different aspects of human alcohol consumption, including chronic ad libitum ethanol feeding through liquid diet and chronic/acute intragastric ethanol administration. The advances from these animal studies help for understanding the molecular and behavioral changes associated with human alcoholism. The accumulated evidence suggests that H_2_S signaling often involves alcohol-caused organ damage (Table 2).

#### 4.2.1. H_2_S Protects from Alcohol-Induced Liver Damage

Many H_2_S-releasing donors have been shown to inhibit alcohol-induced liver damage. In both mouse and rat models of alcohol exposure, the administration of diallyl trisulfide, diallyl disulfide, garlic oil, or garlic polysaccharide significantly limited lipid accumulation and reduced tissue damage [81,82,83,84,85,86]. These H_2_S donors were also able to decrease circulating liver-damage markers, such as alanine aminotranferease and aspartate transaminase [83,84,85,87]. Aldehyde dehydrogenase is a rate-limiting enzyme for ethanol metabolism by changing highly toxic acetaldehyde to nontoxic acetate. Recent evidence suggests the enzymatic activity of aldehyde dehydrogenase could be inhibited in the rat liver by some sulfane sulfur species, such as garlic-derived allyl sulfides, which act as sources for releasing H_2_S slowly in the body [88]. Similar to disulfiram, H_2_S may be used for the treatment of alcohol abuse and alcohol dependence by promoting the accumulation of acetaldehyde in the body.

**Table 2:** Animal models used for analyzing H_2_S signaling and alcohol-induced damage.

Animal Species	Alcohol Administration	H_2_S Supplement	Organ Damage	Target Signaling Pathway	Reference
Kun–Ming mice	50% (*v*/*v*) ethanol, 12 mL/kg, 16 h before sacrifice	30 mg/kg diallyl trisulfide (DATS), orally, for 7 days prior to ethanol	Liver damage	AOE/ROS	Zeng et al. [82]
Sprague–Dawley rats	56% (*v*/*v*) ethanol, 10 mL/kg daily for 8 weeks, gavage	25–100 mg/kg DATS, daily, weeks 5–8	Alcohol fatty liver, steatosis	PPAR-α/SREBP-1c, AOE/ROS, Cas/Bcl2	Chen et al. [83]
Kun–Ming mice	5 g/kg ethanol at 12 h intervals, sacrificed 4 h after third dose, i.g.	25–100 mg/kg diallyl disulfide (DADS), daily, for 7days by gavage	Liver injury, hydropic degeneration	Nrf2/HO-1, ROS, Cas, MAPKs	Zeng et al. [84]
Kun–Ming mice	50% (*v*/*v*) ethanol, 12 mL/kg, sacrificed at 4, 8, and 16 h after exposure	50, 100, 200 mg/kg garlic oil, 2 h before ethanol exposure	Liver injury, steatosis	AOE/ROS, PPAR-α/SREBP-1, FAS, CYP2E1	Zeng et al. [85]
Kun–Ming mice	56% ethanol, 6 mL/kg, orally, for 30 days	150–250 mg/kg garlic power, daily 5 h after ethanol exposure	Liver fibrosis, steatosis	AOE/ROS, TNFα/TGFβ1	Wang et al. [86]
Sprague–Dawley rats	10–12 g/kg ethanol, daily, for 45 days i.g.	200 mg/kg DATS, daily, i.g.	Alcoholic steatohepatitis	CYP2E1, TNFα/Il-4, TGFβ1/Smad	Ronis et al. [87]
Kun–Ming mice	4% ethanol solution as sole source of drinking water freely for 12 weeks	50 µmole/kg NaHS, daily, i.p.	Alcoholic cardiomyopathy	PI3K/AKT, Beclin, TGFβ1/MMP	Liang et al. [89]
Wistar rats	10–30% alcohol per day by i.g for 18 weeks	30 μmole/l NaHS in drinking water	Left ventricular structure and function damage	AOE/ROS, Bax/Bcl2	Zhou et al. [90]
Sprague–Dawley rats	5 mL/kg ethanol, orally	25–50 mg/kg Tert-butylhydroquinone (tBHQ) for 10 days, orally	Gastric ulcers	ROS, COX2, NFkB, Nrf2/HO-1	Rahman et al. [91]
Swiss mice	50% ethanol, 0.5 mL/25 g, gavage, sacrificed 1 h after ethanol	Cysteine (25–100 mg/kg), NaHS (75–300 μmole), or propargylglycine (15–150 mg/kg), gavage, 30 min prior to ethanol	Gastric ulcers	K_ATP_ channels and afferent neurons/TRPV1 receptors	Medeiros et al. [92]
C57BL/6 mice	50% ethanol, 2.5 mL/kg, gavage, sacrificed 1 h after ethanol	l-cysteine or d-cysteine (100 mg/kg) or propargylglycine (100 mg/kg), gavage, 30 min prior to ethanol	Gastric ulcers	d-amino acid oxidase	Souza et al. [93]
Swiss mice	50% ethanol, 0.5 mL/25 g, gavage, sacrificed 1 h after ethanol	150 μmole/kg NaHS; 27 μmole/kg Lawesson’s reagent; 100 mg/kg cysteine; orally, 30 min prior to ethanol	Gastric ulcers	AMPK	de Araújo et al. [94]
Wistar rats	100% ethanol, 1 mL, by gavage, sacrificed at 2 h	15, 50 or 150 μM Na_2_S 30 min prior to ethanol	Gastric ulcers	N/A	Velázquez-Moyado et al. [95]
Wistar rats	100% ethanol, 0.2 mL, by gavage, sacrificed after 30 min	50 mg/kg cysteine, orally	Gastric ulcers	N/A	Velázquez-Moyado et al. [96]
Wistar rats	100% ethanol, 1 mL, by gavage, sacrificed 2 h later	10 mg/kg cysteine; 8.4 mg/kg NaHS; by gavage 2 h prior to ethanol	Gastric ulcers	N/A	Chávez-Piña et al. [97]
Wistar rats	5.25 g/kg ethanol in 27.8 mL/kg milk, by gavage, postnatal day 2–10	1 mg/kg NaHS concurrent with ethanol in milk	Hippocampal apoptosis, impaired spatial memory	BDNF, Brdu, Apoptosis	Mohseni et al. [98]
Wistar rats	5.25 g/kg ethanol in 27.8 mL/kg milk, by gavage, postnatal day 2–10	1 mg/kg NaHS concurrent with ethanol in milk	Reactive gliosis, necrosis and apoptosis of the hippocampus	AOE/ROS, TNF/Il, GFAP, Cas	Mohseni et al. [99]
Sprague–Dawley rats	56% (*v*/*v*) ethanol, 10 mL/kg daily for 8 weeks, gavage	25–100 mg/kg DATS, daily, gavage	Liver damage	AOE/ROS, Cas/Bcl2, CSE	Chen et al. [100]

#### 4.2.2. H_2_S Improves Alcoholic Cardiomyopathy

Alcohol abuse is a major risk factor for the incidence of alcoholic cardiomyopathy and heart disorders. H_2_S is widely reported to provide a significant cardioprotection through its anti-apoptotic, anti-inflammatory, and antioxidant effects [21,22,23]. Accumulated evidence shows that H_2_S improved alcoholic cardiomyopathy. Long-term feeding of the mice with ethanol diet induced irregular arrangement of myocardial fibers and myocardial fibrosis, which could be significantly improved by the administration of H_2_S but deteriorated by PPG treatment. By targeting at miR-21 and miR-221-mediated TGF-β/PI3K/AKT signaling pathways, H_2_S inhibited autophagy-associated proteins (Beclin 1, Atg3, and Atg7) and fibrosis-associated proteins, thus relieving myocardial fibrosis in mice with alcoholic cardiomyopathy [89]. H_2_S has also been shown to protect against chronic alcohol intake-induced left ventricular remodeling in rats via attenuating oxidative stress and apoptosis [90]. 

#### 4.2.3. H_2_S Attenuates Alcohol-Induced Gastric Ulcers

Gastric ulcer is the most common disease caused by alcohol consumption [91]. Accumulated evidence indicated that gastric ulcers by excessive consumption of alcohol can be prevented by many H_2_S donors or activators. Either NaHS or cysteine treatment dose-dependently prevented ethanol-induced macroscopic and microscopic gastric damage in mice by activating K_ATP_ channels and afferent neurons/TRPV1 receptors [92]. Consistent with these findings, Souza et al. also reported that pretreatment with d-cysteine increases the synthesis of H_2_S and protects from ethanol-induced oxidative stress and gastric lesions in mice [93]. AICAR as an AMPK activator prevented ethanol-induced gastric injury in mice by stimulating H_2_S generation [94]. Diligustilide, a dimeric phthalide isolated from *Ligusticum porteri*, is a traditional medicine for treating many diseases including gastric aches. Velázquez-Moyado et al. reported that diligustilide increased gastric H_2_S production and prevented ethanol-induced gastric injury [95]. Carbenoxolone, an anti-ulcer drug, protected from ethanol-induced lesions in rat stomachs via boosting endogenous H_2_S generation [96]. In contrast, Chávez-Piña et al. demonstrated that injection of a CSE inhibitor PPG suppressed H_2_S generation and reversed gastric injury caused by ethanol [97]. The possible explanation may be due to the difference of animal types and doses of ethanol and H_2_S used in each individual study. 

#### 4.2.4. H_2_S Blocks Postnatal Alcohol Exposure-Induced Brain Damage

Maternal alcohol consumption during pregnancy often causes problems in fetal brain development and leads to fetal alcohol spectrum disorder. Mohseni et al. proved that supplementation of H_2_S to rat pups with postnatal ethanol exposure protected from ethanol-induced neurotoxicity and memory loss, mostly due to the proneurogenesis and anti-apoptotic activity of H_2_S [98]. The same group also reported that H_2_S attenuated oxidative–inflammatory cascade and neuronal cell death by ethanol exposure in rat pups during the postnatal period [99]. This evidence suggests that H_2_S is able to improve behavioral and cognitive deficits caused by postnatal alcohol exposure in rat pups. The protective role of H_2_S against brain damage and dysfunction in adult animals with long-term consumption of alcohol need to be further studied. 

### 4.3. Cellular Studies

Alcohol that is mostly metabolized in liver and alcohol consumption often causes liver damage. After incubation with ethanol, human hepatocyte cells exhibited increased apoptosis. It was further found that ethanol inhibited the protein expressions of CSE and CBS, and reduced endogenous H_2_S generation in hepatocyte cells, while diallyl trisulfide boosted H_2_S level and protected against ethanol-induced oxidative stress and apoptosis [100]. After depletion of intracellular cysteine by buthionine sulfoximine, ethanol induced more cell death in human hepatocyte cells, while exogenous glutathione supplementation led to increased cysteine level and cell viability [101]. In another study with rat liver cells, ethanol incubation activated the caspase 3-dependent apoptosis, and the presence of *S*-allyl-l-cysteine was sufficient to prevent cell death induced by ethanol [102]. Consumption of alcohol also leads to adipose tissue inflammation, hyperlipolysis, and apoptosis. Kema et al. reported that diallyl sulfide attenuated ethanol-induced oxidative stress, endoplasmic reticulum stress, and inflammation in 3T3L1 adipocyte cells, and promoted synthesis of M2 phenotype-specific genes in ethanol-exposed RAW 264.7 macrophage cells [103,104]. One study reported that ethanol exposure increased DNA damage of human bronchial epithelial cells, which could be attenuated by diallyl trisulfide pretreatment [105]. These results suggest that H_2_S signaling may be a target for preventing and/or treating ethanol-induced injury of liver and adipose tissues, and possibly lung tissue.

## 5. Prospective

Increasing research has been performed in the discovery of new molecules and mechanisms for assisting in the diagnosis and treatment of diseases associated with cigarette smoking and alcohol abuse. H_2_S is a novel gasotransmitter with great significance in human health. The disrupted H_2_S signaling by cigarette smoking and alcohol drinking has recently come to light. The evidence from cellular and animal studies and also clinical observations identify H_2_S as a regulator of oxidative stress and inflammatory response in the pathogenesis of various diseases associated with cigarette smoking and alcohol drinking. The mechanisms mediating the potential interactions among H_2_S signaling, cigarette smoking, and alcohol abuse in different organs are still incompletely understood. A greater understanding of altered H_2_S signaling can definitely help for developing novel diagnostic approaches. Moreover, the mechanisms underlying the altered expressions and activities of H_2_S-generating enzymes have not been fully clarified. The notion of using fast H_2_S-releasing donors to alter organ dysfunction should be treated with caution. Much work remains to be performed to develop slow H_2_S-releasing donors. Furthermore, more clinical studies with a larger sample size and control subjects can help better characterize the disrupted H_2_S signaling by cigarette smoking and alcohol drinking. There is also a need for accurate measurement of H_2_S level and the activities of H_2_S-generating enzymes in the affected organs by cigarette smoking and/or alcohol usage. Consequently, more research needs to be undertaken to fully determine the potential of targeting H_2_S signaling for prevention and treatment of cigarette smoking- and alcohol-associated diseases.

## Data Availability

Data sharing not applicable.
